# Epidemiological characteristics, clinical manifestations, and treatment outcome of 139 paediatric Ebola patients treated at a Sierra Leone Ebola treatment center

**DOI:** 10.1186/s12879-019-3727-7

**Published:** 2019-01-24

**Authors:** Jia Bainga Kangbai, Christian Heumann, Michael Hoelscher, Foday Sahr, Guenter Froeschl

**Affiliations:** 1 Center for International Health, University of Munich (LMU), Munich, Germany; 2 Department of Environmental Health Sciences, Njala University, Bo, Sierra Leone; 3 Department of Statistics, University of Munich (LMU), Munich, Germany; 4 Division of Infectious Diseases and Tropical Medicine, Medical Center of the University of Munich (LMU), Munich, Germany; 5 Department of Microbiology, College of Medicine and Allied Health Sciences, University of Sierra Leone, Bo, Sierra Leone; 6 34 Military Hospital, Wilberforce, Freetown, Sierra Leone

**Keywords:** Ebola, Ebola treatment center, Paediatric, Treatment outcome, Sierra Leone

## Abstract

**Background:**

The West Africa Ebola Virus Disease (EVD) outbreak in 2014–2016 was declared by the World Health Organization (WHO) a public health emergency of international concern. Most of the previous studies done in Sierra Leone relating to the clinical and epidemiological features of EVD during the 2014–2016 West African outbreak focused on adult EVD patients. There have been conflicting reports about the effects of EVD on children during previous outbreaks.

**Methods:**

This is an observational retrospective analysis of medical data of all laboratory confirmed paediatric EVD patients below 15 years of age who were admitted at the 34 Military Hospital Ebola Treatment Center (ETC) in Wilberforce, Sierra Leone between June 2014 to April 2015. We analyzed the sociodemographic and clinical characteristics of paediatric EVD cases contained in case report forms that were collected by Ebola surveillance officers and clinicians at the 34 Military Hospital ETC. Both univariate and multivariate logistic regression models were used to determine the sociodemographic and clinical characteristics of paediatric EVD patients that were associated with EVD facility-based mortality.

**Results:**

The majority of the paediatric EVD cases in this study were female (56.1%), pupils (51.1%), and 43.2% belonged to the age group between 10 years and below 15 years. The median age of the paediatric EVD cases was 9 years (interquartile range = 4 to 11 years). Adjusting for other covariates in the model, male paediatric EVD patient (AOR = 13.4, 95% CI = [2.07–156-18], *p* <  0.05), EVD patient with abdominal pain (AOR = 11.0, 95% CI = [1.30–161.81], *p* <  0.05), vomiting (AOR = 35.7, 95% CI = [3.43–833.73], *p* <  0.05), signs of conjunctivitis (AOR = 17.4, 95% CI = [1.53–342.21], *p* <  0.05) and difficulty in breathing (AOR = 23.3, 95% CI = [1.92–713.01], *p* <  0.05) at the time of admission had increased odds of dying during EVD treatment.

**Conclusions:**

We recommend the adoption of case definitions currently in vigour to cater for specific characteristics of paediatric patients. Subgroups that can be identified by applying the model developed in this study may require special attention and intensified care.

## Background

Ebola virus disease (EVD) is a severe infectious disease that was discovered in the Democratic Republic of Congo in 1976 [1–3]. The virus that causes EVD belongs to the Filoviridae family [1, 4]. An EVD outbreak in West Africa which was detected in March 2014 prompted the World Health Organization (WHO) to declare it a “public health emergency of international concern” [5, 6]. According to the WHO there were ultimately more than 28,000 cases and more than 11,000 deaths in the course of the West African Ebola outbreak [7]. Humans become infected with EVD by coming into direct contact with infected human bodily fluids, or the bodily fluids or organs of infected bush animals such as bats, monkeys and chimpanzees [5]. Sierra Leone which was also heavily affected [5] by the West African EVD outbreak recorded its first case in May 2014 [8]. Most studies relating to the clinical and epidemiological features of EVD at the time of the West African outbreak focused on adult EVD patients. WHO Ebola Response Team [5], Schieffelin et al. [9], Lado et al [10] and Agua-Agum J, et al [11] have published extensive details of the clinical, laboratory and epidemiological characteristics of mixed cohort of patients affected by the West African EVD outbreak. Mostly biased by the WHO case definition for a suspected Ebola case, the majority of the patients affected in Sierra Leone by the West African outbreak like others in previous ones were characterized by fever, fatigue, muscle pain, headache, and sore throat, vomiting, diarrhea, rash, kidney, liver function failure, sometimes bleeding (although to a lesser extent than previously known), and an incubation period of 2–21 days (median, 14 days) [1, 2, 5, 12].

There have been conflicting reports about the effects of EVD on children during EVD outbreaks. McElroy AK, et al. reported a Case Fatality Rate (CFR) of 100% (for children EVD cases for whom serum were available), and 28.6% for children < 5 years and 6–15 years respectively for the 2000–2001 EVD outbreak in Gulu district, Uganda [13]. Different studies reported a moderate CFR (57.1%) for paediatric EVD cases during the 2014–2016 EVD outbreak in Sierra Leone [14, 15]. Peacock et al. reported data for children EVD cases from different outbreaks [16] which tend to suggests that the proportion of EVD infected children varies for different settings and over time. There were more (79.0%) paediatric EVD cases compared to EVD cases belonging to the age group of 15 years and above during the 2000–2001 EVD outbreak in Uganda [13]. Leligdowicz A et al. however reported a lower (19%) EVD cases for children compared to cases 15 years and above for the 2014–2016 West African EVD outbreak [17]. Also, the proportion of children involved in the 1995 EVD outbreak in Zaire was 27/315 (9%), 90/218 (41%) for the 2000–2001 Sudan strain EVD outbreak in Gulu, Uganda, and 147/823 (18%) for the 4 most affected countries in the 2014–2016 EVD outbreak in West Africa [18]. The World Health Organisation (WHO) reported increasing incidence of EVD cases with increasing age among children in Liberia and Sierra Leone but this pattern was not observed for children in Guinea [11]. Previous papers have claimed that children were relatively spared during EVD outbreaks [11, 19]; which portrays that there is variation in the epidemiology, clinical features and outcomes of paediatric EVD cases [20]. Skrips LA, et al. attributed the high incidence of paediatric EVD cases to the less restriction of contact by children coupled with their limited or non-existent understanding of the mechanisms of EVD transmission dynamics [21]. Athena P and colleagues however attributed the small percentage of global EVD cases for children to cultural practices [22]. In spite of claims that children had reduced risks of infection as compared to adults [19, 23] in previous EVD outbreaks, and that paediatric EVD cases were underreported during the 2014–2016 West African EVD outbreak [13, 24], the West African EVD outbreak recorded a high CFR in children younger than 5 years [9, 11, 25]. Cumulative CFR for the West African EVD outbreak varies but was approximated at 40% [5] with young children and older adults having the highest CFRs [9]. There are reports of disproportionate CFRs among children affected by the 2014–2016 West African outbreak [9, 26]. Schieffelin J.S et al. reported a cumulative CFR of 73.4, 66.1 and 80.4% for children < 15 years, 15–44 years and for EVD patients > 45 years respectively [9]. One Ugandan study involving 55 confirmed EVD cases infected with Sudan Virus Ebola strain reported a high survival rate for children compared to adult [13].

Clinical data and studies on paediatric EVD treatment outcomes relating to the West African EVD outbreak are scarce, often with small sample sizes [25] and largely focused on reported symptoms by EVD patients on arrival for admission [1, 2, 5, 9, 12, 25, 27]. For previous EVD studies, the number of paediatric EVD patients in a cohort investigated ranges between 20 and 55 laboratory-confirmed EVD cases [13, 23]. Kourtis A. P et al [22] argued that the fewer studies involving paediatric EVD cases can be attributed to the fact that less number of children become infected with EVD compared to adults due to their lower risk of exposure to EVD which includes caring for EVD sick patients-either in a healthcare setting or at home, and or handling the remains of persons who have died of EVD.

The CFR in EVD in neonates is generally higher than in children in other age groups. All neonates born to previously infected EVD mothers died within 19 days after birth during the 1976 outbreak in Zaire [28] and subsequent outbreaks have also confirmed such observations [29].

There is no approved EVD treatment or vaccine against EVD [6, 30] but supportive care and management by intravenous fluids intake proved to be crucial for EVD patient survival during the 2014–2016 outbreak [6, 30]. However currently there are series of experimental therapies and vaccines including brincidofovir [31], ZMapp [18], TKM 130803 [32], Favipiravir [33], the monoclonal antibody MAb114 [34], and convalescent plasma of EVD patients [35] that has been approved by the WHO for use during outbreaks on compassionate ground.

In this study we describe the epidemiological characteristics, clinical manifestations and treatment outcome of 139 laboratory-confirmed paediatric EVD patients below 15 years of age who were admitted at the 34 Military Hospital Ebola Treatment Center (ETC) in Wilberforce, Sierra Leone between June 2014 to April 2015. We also determine the factors that are associated with EVD treatment outcomes of these EVD confirmed paediatric EVD cases using a large dataset. Early studies that investigated paediatric EVD cases were faced with many limitations including small sizes, incomplete patient information, selection and lead time biases. The main strength of this study is our large sample size of paediatric EVD cases belonging to the age group 0 – below 5 years coupled with the fact that our data came from an operational and hence reflect the ground reality.

## Methods

### Study design

Our study is an observational retrospective study that included all laboratory confirmed EVD patients below 15 years of age who were admitted at the 34 Military Hospital ETC situated in Wilberforce section of Freetown in Sierra Leone between June 2014 to April 2015.

These confirmed paediatric EVD patients were brought to the 34 Military Hospital triage center that was located at the Accident and Emergency Department by their parents or relatives because they were referred by EVD surveillance health workers of the National Ebola Response Surveillance Team, self-referred after coming in contact with a suspected or confirmed Ebola case, or because they presented with key Ebola signs and symptoms such as fever, headache, joint pain, diarrhoea, vomiting, or and bleeding [1, 2, 5, 9, 10, 12]. All EVD paediatric patients were first screened by trained clinicians against the WHO definition for a suspected EVD case [28] prior to EVD laboratory confirmation testing. Ebola is classified in three clinical stages: Stage one EVD which is also known as the dry or early phase is characterised by the absence of vomiting, diarrhoea, or organ dysfunction; Stage two which is also referred to as the wet phase is characterised by vomiting and diarrhoea; and Stage three or the organ dysfunction phase of which human organ failure is the most prominent feature.

For all paediatric EVD cases laboratory confirmation tests were done using real-time quantitative reverse transcriptase polymerase-chain-reaction (qRT-PCR) method at the National Public Health Laboratory at Lakkah in Freetown, Sierra Leone.

### EVD treatment protocol

All laboratory-confirmed EVD cases in this study were routinely provided oral rehydration salts with dose dependent on the severity of the dehydration of the paediatric EVD patient; intravenous lactated Ringer’s solution and other supplements to correct for electrolyte imbalance; acetaminophen or ibuprofen for muscle pain and headache, anti-infective ciprofloxacin or cefixime, and the anti-malaria drug naphthoquine phosphate tablets. The antacid drugs ranitidine or omeprazole were given to patients experiencing upper abdominal pain. EVD treatments in this study were performed in accordance with the World Health Organisation (WHO) protocol of urgent interim guidance for EVD case management for viral haemorrhagic fever [30]. The treatment method in the other ETCs that were operating in Sierra Leone during the 2014–2016 EVD outbreak was mostly supportive and mostly included maintaining electrolyte balance in EVD patients.

### Sierra Leone health infrastructure

Sierra Leone is located in West Africa. There is one government referral hospital in each of the 5 provinces or national areas. The rural areas of Sierra Leone are also served by several district health hospitals (DHHs), community health centers (CHCs) and community health posts (CHPs). All government referral hospitals and some DHHs served as either an ETC or an Ebola Holding Center (EHC) during the 2014–2016 Ebola outbreak. During the EVD outbreak several hospitals and health care facilities that were run by foreign organizations also operated ETCs. The 34 Military Hospital which provided data for this study is a 150-bed hospital located in the capital city Freetown. The hospital which is headed by a Brigadier Surgeon General is operated by medical doctors and paramedics that are attached to the 34th Military Battalion of the Sierra Leone Armed Forces (SLAF).

### Ethics review

The Sierra Leone Ethics and Scientific Review Committee (Opinion Date March 29, 2017) and the Institutional Review Board at the Ludwig-Maximilians-Universität in Munich, Germany (Opinion No. LMU 17–582) approved this study. The Sierra Leone Ethics and Scientific Review Committee provided ethical clearance for conducting this study and waived the requirement to obtain informed consent on the grounds that this is an observational retrospective study on patients in charge in a medical facility under circumstances that did not allow at that time for individualized informed consent, and that data is resented in an aggregate manner focusing on outcome in one entire facility.

### Data collection and processing

At the 34 Military Hospital ETC trained clinicians and Ebola surveillance officers compiled on hard copies of CRF the medical history containing demographic, laboratory and clinical information of all suspected paediatric EVD patients who presented themselves with key signs and symptoms associated with EVD. We later transferred the medical data of all laboratory confirmed paediatric EVD patients from the CRF to a Microsoft Excel (Microsoft, Redmond, Washington, USA) [36] form for both descriptive and analytical statistics processing. The medical data of confirmed paediatric EVD patients included both clinical (whether patient had fever, headache, joint pain, anorexia, muscle pain, chest pain, abdominal pain, cough, diarrhoea, vomiting, fatigue, bleeding, skin rash, difficulty in swallowing or breathing, conjunctivitis and being in a confused state at the time of admission) and demographic data (age group, sex, education level). This study analysed the anonymized medical data of 139 paediatric EVD patients. The data were anonymized by Ebola surveillance data entry clerks and clinicians attached at the 34 Military Hospital in Freetown, Sierra Leone. The anonymized data were later stored in secured computer files at the 34 Military Hospital in Freetown, Sierra Leone.

### Statistical analysis

R software package version 3.3.1 [37] was used for all data analyses; the source codes are available upon request. A *p*-value < 0.05 was considered as statistical significance for all two-sided statistical tests. We present as frequencies, proportions, means (standard deviations) and medians (interquartile ranges) the outputs of descriptive analysis and used Fisher’s Exact test to compare proportion of various variables. Both univariate and multivariate logistic regression model were used to determine the clinical and non-clinical characteristics of paediatric EVD patients that were associated with EVD in-facility mortality. To understand the association between education and in-facility mortality (CFR) we grouped the paediatric EVD patients into two; no-education and education groups. The education group is comprised of paediatric EVD cases with either primary or secondary education while the paediatric EVD patients in the no-education group have no education experience. We later used the Receiver Operating Characteristic Curve (ROC) to determine our logistic model’s ability to predict whether a paediatric EVD patient will be cured given certain clinical and sociodemographic characteristics of a patient. We then calculated the Area Under the Curve (AUC) value obtained from the ROC curve to determine the accuracy of the model to predict paediatric EVD patient treatment outcome.

## Results

### Descriptive characteristics of cases

Between June 2014 to April 2015, 1076 confirmed EVD cases, of which 139 (12.9%) were paediatric cases below 15 years of age, were admitted at the 34 Military Hospital ETC for EVD treatment. January 2015 recorded the highest number of confirmed EVD cases to be admitted at the 34 Military Hospital ETC, with 326 patients in total admitted, of which 52 (16.0%) were paediatric cases.

### Demographic factors

The majority of the paediatric EVD cases were female (78/139, 56.1%), pupils (71/139, 51.1%), and (60/139, 43.2%) belonged to the age group between 10 years and below 15 years (Table 1). The median age of the paediatric EVD cases was 9 years (interquartile range = 4 to 11 years).

**Table 1:** Sociodemographic factors, treatment outcome and case fatality rates of paediatric EVD patients treated at the 34 Military Hospital in Sierra Leone during the 2014–2016 EVD outbreak

EVD patients’ sociodemographic Characteristics	N (%)	Survived N (%)	Died N (%)	Case fatality rate (%)	*p*-value*
Total	139 (100)	108 (77.7)	31 (22.3)	22.3	
Female	78 (56.1)	68 (63.0)	10 (32.3)	12.8	< 0.05
Male	61 (43.9)	40 (37.0)	21 (67.7)	34.4	
0 to < 5 years	37 (26.6)	23 (21.3)	14 (45.2)	37.8	< 0.05
5 to < 10 years	42 (30.2)	31 (28.7)	11 (35.5)	26.2	
10 to < 15 years	60 (43.2)	54 (50.0)	6 (19.4)	10.0	
No education	37 (26.6)	23 (21.3)	14 (45.2)	37.8	< 0.05
Primary education	71 (51.1)	54 (50.0)	17 (54.8)	23.9	
Secondary education	31 (22.3)	31 (28.7)	0 (0.0)	0	

_*_p-value was obtained by applying chi square test by comparing the case fatality rates and sociodemographic characteristics of paediatric EVD patients

### Case fatality rate

The overall CFR among the admitted 139 confirmed paediatric EVD patients was 22.3% (31/139). One hundred and eight out of 139 (77.7%) paediatric EVD patients were discharged alive from the 34 Military Hospital ETC after treatment. There was a statistically significant association between gender, age groups and education levels and the CFR for paediatric EVD cases. Male paediatric patients had higher (34.4%) CFR than female (CFR = 12.8%, *p* <  0.05). There was a negative correlation between paediatric EVD patient age and CFR. The CFR for paediatric EVD patients below 5 years of age was higher (CFR = 37.8%, *p* <  0.05) than those of patients between 5 years to less than 10 years of age (CFR = 26.2%); and 10 years to less than 15 years of age (CFR = 10.0%).

The CFR for paediatric EVD patients with no education was higher (CFR = 37.8%, *p* <  0.05) compared to those at primary level education (CFR = 23.9%). All paediatric EVD patients with secondary level education who were treated in this study were released alive after treatment.

### Clinical symptoms

The majority of the paediatric EVD cases at the time of admission had anorexia (99.1%), chest pain (98.6%), muscle pain (97.8%), headache (95.0%), fever (82.7%), diarrhoea (71.3%), fatigue (67.0%), Stage 2 EVD infection (64.0%) and abdominal pain (59.7%) when they reported at 34 Military Hospital ETC for admission (Table 2). There was a statistically significant association between EVD paediatric patients with diarrhoea, vomiting, fatigue, skin rash, bleeding, difficulty in swallowing, conjunctivitis, difficulty in breathing, Stage 2 and 3 EVD infections compared to those without these characteristics. All paediatric EVD patients with skin rash at the time of admission died during treatment (CFR = 100%, *p* = 0.05) compared to 21.2% of paediatric EVD patients without skin rash at the time of admission who died during treatment. Paediatric EVD patients with Stage 3 EVD infection (CFR = 81.3%, *p* <  0.05), difficulty in breathing (CFR = 76.9%, *p* <  0.05), bleeding (CFR = 70.0%, *p* <  0.05), difficulty in swallowing (CFR = 56.5%, *p* <  0.05), conjunctivitis (CFR = 50.0%, *p* <  0.05), vomiting (CFR = 40.4%, *p* <  0.05), fatigue (CFR = 30.1%, *p* <  0.05), diarrhoea (CFR = 28.3%, *p* <  0.05), abdominal pain (CFR = 26.5%, *p* = 0.21) and anorexia (CFR = 22.5%, *p* = 1) at the time of admission have higher CFR compared to paediatric patients who did not report vomiting, fatigue, bleeding, difficulty in swallowing, difficulty in breathing, conjunctivitis, anorexia, abdominal pain, Stage 3 EVD infection or diarrhoea at the time of admission.

**Table 2:** Clinical symptoms, treatment outcome and case fatality rates of paediatric EVD patients treated at the 34 Military Hospital in Sierra Leone during the 2014–2016 EVD outbreak

EVD patients clinical symptoms	N (%)	Survived N (%)	Died N (%)	Case fatality rate (%)	*p*-value*
Total	139 (100)	108 (77.7)	31 (22.3)	22.3	
Fever	115 (82.7)	90 (82.3)	25 (80.7)	21.7	0.79
Headache	132 (95.0)	103 (95.4)	29 (93.6)	22.0	0.65
Anorexia	138 (99.3)	107 (99.1)	31 (100.0)	22.5	1.00
Muscle pain	136 (97.8)	107 (99.1)	29 (93.6)	21.3	0.13
Chest pain	119 (98.6)	96 (88.9)	23 (74.2)	19.3	0.08
Abdominal pain	83 (59.7)	61 (56.5)	22 (71.0)	26.5	0.21
Cough	67 (48.0)	54 (50.0)	13 (41.9)	19.4	0.54
Diarrhoea	99 (71.3)	71 (65.7)	28 (90.3)	28.3	< 0.05
Vomiting	57 (41.0)	34 (31.5)	23 (74.2)	40.4	< 0.05
Fatigue	93 (67.0)	65 (60.2)	28 (90.3)	30.1	< 0.05
Skin rash	2 (1.4)	0 (0.0)	2 (6.5)	100.0	0.05
Bleeding	10 (7.2)	3 (2.8)	7 (22.6)	70.0	< 0.05
Difficulty swallowing	23 (16.6)	10 (9.3)	13 (41.9)	56.5	< 0.05
Conjunctivitis	20 (14.4)	10 (9.3)	10 (32.3)	50.0	< 0.05
Difficulty breathing	13 (9.4)	3 (2.8)	10 (32.3)	76.9	< 0.05
Stage one EVD infection	34 (24.5)	34 (31.5)	0 (0.0)	0.0	< 0.05
Stage two infection	89 (64.0)	71 (65.7)	18 (58.1)	20.2	
Stage three EVD infection	16 (11.5)	3 (2.8)	13 (41.9)	81.3	

*p-value was obtained by applying chi square test by comparing the case fatality rates and clinical characteristics of paediatric EVD patients

Paediatric EVD patients who reported fever (CFR = 21.7%, *p* = 0.79), headache (CFR = 22.0%, *p* = 0.65), muscle pain (CFR = 19.3%, *p* = 0.13) and chest pain (CFR = 19.3%, *p* = 0.08) at the time of admission have reduced CFR as compared to paediatric patients who did not report fever, headache, muscle pain or chest pain at the time of admission.

### Multivariate analysis of facility based mortality

Our stepwise multivariate logistic regression analysis shows that male paediatric EVD patient, paediatric EVD patient who vomited, had abdominal pain, difficulty in breathing, and conjunctivitis at the time of admission were the most important factors associated with paediatric EVD facility based mortality. There were differences in the likelihood that a paediatric EVD patient will die during EVD treatment related to gender difference, or whether EVD patient reported abdominal pain, difficulty in breathing, conjunctivitis or vomiting at the time of admission. Holding other covariates in the model constant, male paediatric EVD patient (AOR = 13.4, 95% CI = [2.07–156-18], *p* <  0.05), abdominal pain (AOR = 11.0, 95% CI = [1.30–161.81], *p* <  0.05), vomiting (AOR = 35.7, 95% CI = [3.43–833.73], *p* <  0.05), conjunctivitis (AOR = 17.4, 95% CI = [1.53–342.21], *p* <  0.05) and difficulty in breathing (AOR = 23.3, 95% CI = [1.92–713.01], *p* <  0.05) had increased odds of dying during EVD treatment (Table 3).

**Table 3:** Multivariate logistic regression output of paediatric EVD patients sociodemographic and clinical factors associated with treatment outcomes

Patient symptoms	Crude OR	95% CI	Adjusted OR	95% CI
Sex-Male	3.57	1.56–8.64	13.36	2.07–156.18
Age group in years (5 to < 10)	1.72	0.66–4.54	0.55	0.06–4.74
Age group in years (10 to < 15)	5.48	1.95–17.16	0.16	0.01–3.02
Education	3.36	1.76–6.82	0.15	0.01–1.04
Fever	0.83	0.31–2.50	1.66	0.25–14.17
Headache	0.70	0.14–5.09	0.05	0.001–1.90
Chest pain	0.70	0.14–5.09	0.10	0.01–1.04
Abdominal pain	1.88	0.82–4.66	11.01	1.30–161.81
Cough	0.72	0.32–1.61	0.68	0.12–3.68
Diarrhoea	4.86	1.59–21.28	0.99	0.14–7.19
Vomiting	6.26	2.63–16.26	35.65	3.43–833.73
Fatigue	6.17	2.03–26.94	3.18	0.42–33.99
Bleeding	10.21	2.64–50.09	2.78	0.31–26.74
Difficulty swallowing	7.08	2.72–19.09	0.26	0.02–2.94
Conjunctivitis	4.67	1.72–2.82	17.38	1.53–342.21
Difficulty breathing	16.67	4.66–79.19	23.28	1.92–713.01

Table 3 shows the output of a univariate and a multivariate analysis of paediatric EVD patients sociodemographic and clinical variables associated with EVD treatment outcomes. The crude OR is obtained by a logistic regression model with only that one variable as predictor. The adjusted OR is obtained from a multivariate logistic regression model, starting with all available sociodemographic and clinical predictors, after a stepwise backward elimination using the Akaike Information Criterion (AIC)

**Fig. 1:**
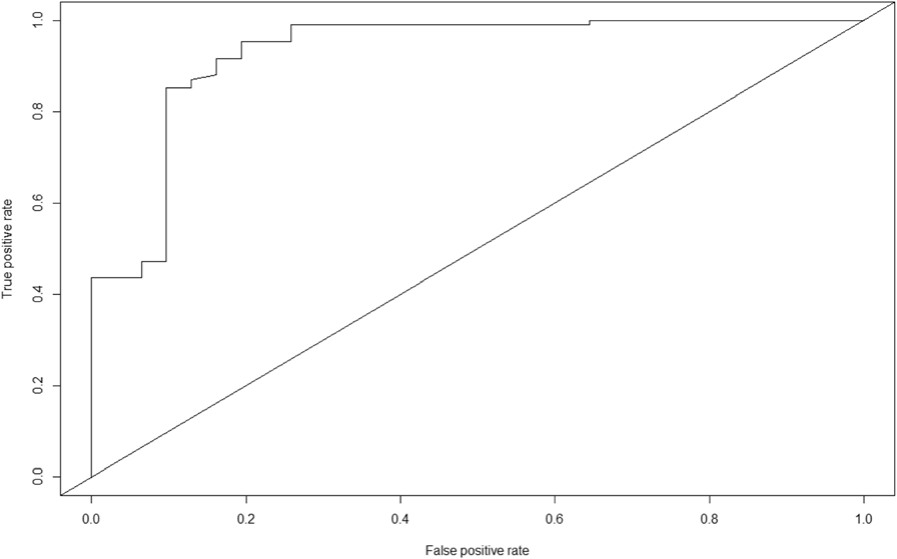
ROC Curve on determinants and treatment outcome. The ROC curve shows that the model after stepwise logistic regression selection has a high capacity to discriminate paediatric EVD patient treatment outcome using their clinical and demographic characteristics

Our ROC shows that the model including the characteristics sex, abdominal pain, vomiting, conjunctivitis and difficulty in breathing has a high discriminative capability for selecting paediatric EVD cases who will be cured during treatment with an AUC of 0.94 (Fig. 1).

## Discussion

The West African EVD outbreak in 2014–2016 recorded the highest number of incidence and mortality rates since the discovery of Ebola virus in 1976 [5, 22]. Our study described the treatment outcomes of 139 laboratory-confirmed paediatric EVD patients admitted at the 34 Military Hospital in Wilberforce, Sierra Leone between June 2014 to April 2015. Paediatric EVD cases in previous outbreaks have been assumed to be underreported because of outbreak dynamics and societal structure [13]. Our study reported a slightly higher proportion of female (56.1%) EVD cases compared to male which is consistent to one Ugandan study [23]. The Ugandan study which analyzed data from an EVD outbreak in Gulu district attributed the high incidence of female paediatric EVD cases to household chores given to female children such as providing patient care in the home. This traditional role of the girl child is also common in Sierra Leone. Compared to other age groups in our study, paediatric EVD patients below 5 years of age recorded the highest CFR (37.8%); which is similar to findings in other studies [8, 13, 38]. One mixed cohort study involving adult and paediatric EVD cases reported a high CFR among young children and elderly EVD patients in the 2014–2016 West African EVD outbreak [5]. Another Ugandan study that investigated the 2000–2001 Sudan virus strain EVD outbreak in Gulu district reported a CFR of 76.9 and 37.5% for 55 paediatric laboratory-confirmed EVD patients below 5 years; and between 6 years to below 15 years of age respectively [16]. The relatively higher CFR among paediatric EVD patients below 5 years in our study is associated to the shorter incubation period of the disease in this set of patients. As reported in a previous study, EVD has a shorter incubation period among paediatric cases below 10 years of age [39]. Our overall CFR (22.3%) was substantially lower compared to the CFR for the 13 previous Zaire EVD outbreaks combined (81.0%) [40] as well as for the average CFR (71.0%) computed by the WHO for mixed age groups for the 2014–2016 West African EVD outbreak [5]. However, it has to be kept in mind that our study is reporting facility based CFRs. Some studies associated the high case fatalities in previous Zaire EVD outbreaks with clinical determinants such as multiple foci of hemorrhage [28, 29].

Our higher CFR for paediatric EVD patients with no education compared to those with primary and secondary levels education may not be unconnected to the role played by health workers and school authorities in raising awareness and sensitizing school children about the transmission methods and effects of Ebola during the 2014–2016 outbreak. Both primary and secondary schools pupils benefited from daily health education programs dealing with the signs and symptoms, transmission methods, preventive and control measures of Ebola. Early identification of EVD signs and symptoms backed by early treatment increases one’s odds of surviving EVD treatment. EVD patients that report early for treatment experience less severe presentation at the time of diagnosis compared to those who report late. T.E.C. Jones-Konneh et al. reported that the expert knowledge and skills of health practitioners made the difference in controlling and reducing the impact of the Ebola epidemic in Sierra Leone [41]. Another Sierra Leone study by Stehling-Ariza T and colleagues attributed the quicker identification of suspected Ebola cases as well as the interruption of Ebola transmission to active case surveillance and health education during the outbreak period [42].

The majority of the paediatric EVD cases in our study reported fever, headache, anorexia, muscle pain, chest pain, abdominal pain, diarrhoea, fatigue and Stage 2 EVD infection at the time for admission at 34 Military Hospital ETC. Elhadji Ibrahim Bah et al. [30], Olupot-Olupot [31], and Theocharopoulos et al. [32] had similar findings for mixed age groups but excluding Stages of EVD infection for patients investigated during 2014–2016 West African outbreak. Elhadji Ibrahim et al. described 37 laboratory-confirmed mixed cohort EVD patients with median age of 38 years, majority (65.0%) of whom were men, fever (84.0%), fatigue (65.0%), and diarrhea (62.0%) with a CFR of 43.0% [30]. In his review Olupot-Olupot noted that typical paediatric EVD symptoms for cases less than 12 years of age include mostly fever, weakness, loss of appetite, profuse diarrhea, vomiting and bleeding; in older children headache, backache, chest pain and abdominal pain are playing a more prominent role [31]. Theocharopoulos G et al. studied 249 confirmed mixed-cohort of EVD cases with a 45.0% CFR of which malaise (90.0%), fever (83.0%), diarrhoea (63.0%), headache (73.0%) and vomiting (60.0%) were the most common symptoms. Considering the fact that EVD is a disease with non-specific symptoms, these can pose as a dilemma in EVD outbreak foci because paediatric EVD clinical features are similar to those of other common childhood infections. In order to mitigate a potentially high risk of nosocomial infections in non-EVD cases that present themselves with symptoms compliant with EVD case definitions in vigor, criteria that are discriminative in terms of both probability of true positive cases and of level of adverse outcome would serve as a valuable individualized risk assessment. Some of our findings on the clinical symptoms of paediatric Ebola cases are different from those for adult EVD cases. Barry et al. recorded a high (60.0%) proportion of adult EVD cases with vomiting compared to ours (41.0%); as well as a statistically significant increase in the odds of dying for adult EVD cases who presented with bleeding at the time of admission (*p* = 0.001) [43]. Our study reported that the odds of dying from bleeding for paediatric EVD cases were not statistically significant (*p* > 0.05). Ohuabunwo et al. reported a high (30.0%) proportion of adult EVD cases with bleeding [44]; ours was 7.2%. Barry et al. also reported lower proportions for diarrhoea (34.0%) and muscle pain (23.0%) for adult Ebola cases [43] compared to ours for paediatric EVD cases (diarrhoea = 71.3%, muscle pain = 97.8%). The proportion of adult EVD cases presenting with anorexia reported by Ohuabunwo et al. was also lower (55.0%) [44] than ours (99.3%). We recorded a higher (98.6%) proportion of paediatric EVD cases who presented with chest pain than those reported by Dallatomasina S et al. (44.0%) for adult EVD cases [2]. We also reported a 100% CFR (*p* = 0.05) for paediatric EVD patients who presented with skin rash (maculopapular rash) at the time of admission but this feature was not prominent among adult EVD cases during the 2014–2016 West African EVD outbreak [45].

One limitation of our study is the lack of follow up to determine the outcome of paediatric EVD cases that were released alive which may have revealed late mortality. Additionally, considering that our medical records did not capture the viral load of EVD patients at the time of their admission and the date of EVD onset as determined by the appearance of EVD signs and symptoms, we were thus unable to determine the effect of treatment delay and viral load on EVD treatment outcome. The findings of our facility-based EVD patient treatment outcomes have to be seen in the context of a specialized treatment facility that was located in the heart of a country’s capital, therefore the potential external validity of our findings has to be taken with caution.

Our logistic model has an ROC with a high AUC of 0.94 to discriminate between paediatric EVD patients who were cured from those who died during treatment by using the characteristics sex of the patient, reported abdominal pain, vomiting, difficulty in breathing or showing signs of conjunctivitis at the time of admission. An individual’s high risk of dying as implied by our model would as a consequence justify prompt and intensified treatment, which may be a scarce resource during peak periods of an ongoing outbreak.

## Conclusions

Our study identified both epidemiological and clinical features that were associated with EVD infection, CFRs as well as those that are significant predictors for paediatric EVD treatment outcome. We reported that slightly more females were infected with EVD compared to males and that EVD cases below 5 years of age, as well as those cases that reported difficulty in breathing, difficulty in swallowing, signs of conjunctivitis and those with Stage 3 EVD infection at the time of admission recorded higher CFRs compared to the other paediatric EVD cases without these criteria. Additionally, we observed that male paediatric EVD patients, paediatric EVD patient who reported abdominal pain, difficulty in breathing, vomiting and showed signs of conjunctivitis at the time of admission tended to have increased odds of dying during EVD treatment. Our model suggests an adapted set of criteria for case definitions that would allow a differentiated approach to clinical management that can be assumed to be beneficial to a subgroup of paediatric patients at high risk of dying in the course of treatment. We are also suggesting the formulation of a separate paediatric EVD case definition to handle the dissimilarities in CFRs and clinical symptoms between childhood EVD cases and adult EVD cases and to facilitate discrimination from other childhood diseases that have similar clinical symptoms like those of paediatric EVD.

## Abbreviations

AOR: Adjusted odds ratio
AUC: Area under the curve
CFR: Case fatality rate
CI: Confident interval
ETC: Ebola treatment center
EVD: Ebola virus disease
OR: Odds ratio
qRT-PCR: quantitative reverse transcriptase polymerase-chain-reaction
ROC: Receiver operating characteristic curve
WHO: World health organization
